# Colorimetric Detection of Salicylic Acid in Aspirin Using MIL-53(Fe) Nanozyme

**DOI:** 10.3389/fchem.2020.00671

**Published:** 2020-09-18

**Authors:** Ling Liang, Yaojing Huang, Wenren Liu, Weiyuan Zuo, Fanggui Ye, Shulin Zhao

**Affiliations:** State Key Laboratory for the Chemistry and Molecular Engineering of Medicinal Resources, College of Chemistry and Pharmaceutical Science of Guangxi Normal University, Guilin, China

**Keywords:** aspirin, complexation, colorimetric detection, MIL-53(Fe), nanozyme, salicylic acid

## Abstract

The impurity of salicylic acid (SA) in aspirin is a required inspection item for drug quality control. Since free SA is significantly toxic for humans, the content determination of free SA is absolutely necessary to ensure people's health. In this work, a facile colorimetric method was developed for the detection of SA in aspirin by utilizing the MIL-53(Fe) nanozyme. As MIL-53(Fe) possesses enzyme mimicking catalytic activity, 3,3,5,5-tetramethylbenzidine (TMB) can be easily oxidized to blue-oxidized TMB (oxTMB) with the existence of H_2_O_2_. Moreover, an inhibition effect on the catalytic activity of the MIL-53(Fe) nanozyme is induced due to the specific complexation between SA and Fe^3+^ in the center of MIL-53(Fe), which results in a lighter color in the oxTMB. The color change of oxTMB can be seen easily by the naked eye with the addition of different concentrations of SA. Thus, a simple colorimetric platform was established for effectively monitoring SA. A good linear relationship (*R*^2^ = 0.9990) was obtained in the concentration range of 0.4–28 μmol L^−1^, and the detection limit was 0.26 μmol L^−1^. In particular, the rationally designed system has been well-applied to the detection of SA impurity in aspirin. Satisfyingly, the detection results are highly in accord with those of HPLC. This novel colorimetric platform broadens the application prospects of nanozymes in the field of pharmaceutical analysis.

## Introduction

Salicylic acid (SA), as a major phytohormone, generally plays an important role in regulating diverse physiological processes such as defense responses, thermogenesis, and germination (Wang et al., 2010; Marques et al., 2020). SA is a promising material and it is commonly exploited in the manufacture of pharmaceutical products (Kopp and Ghosh, 1994). Additionally, SA is widely used in the cosmetics industry as an organic acid (Chanakul et al., 2016). Thus, SA has broadened application prospects in many fields. However, SA is one of the common pollutants in industrial wastewater (Collado et al., 2010). More importantly, SA may cause some toxic side effects on the human body. Because of its relatively strong acidity, SA can not only stimulate and damage the mouth, esophagus, and gastric membrane, but can also invoke adverse symptoms such as metabolic disorders, ototoxicity, fetal malformations, and central nervous system depression (Tian et al., 2009). Aspirin, as a widely used drug, has the effects of an antipyretic, analgesic and anti-inflammatory (Dahl and Kehlet, 1991; Parham and Rahbar, 2009). However, SA can be easily produced due to the incomplete acetylation in the production process of aspirin or hydrolysis during the refining process and storage. Besides, the phenolic hydroxyl group in the free SA is readily oxidized and consequently form a series of colored quinone compounds, leading to discoloration of aspirin. There have corresponding safety limits for free SA in aspirin in countries around the world. From the above consideration, the contents of SA need to be frequently determined to ensure people's health.

There have been many analytical strategies for the detection of SA so far. For instance, one common analysis method is the spectrophotometric Trinder test, which is based on the formation of a coordination complex between SA and Fe^3+^ (Shokrollahi et al., 2017). Beyond that, many chromatography-based methods like high-performance liquid chromatography (HPLC) (Aboul-Soud et al., 2004; Croubels et al., 2005), gas chromatography (GC) (Tanchev et al., 1980), HPLC-MS/MS (Uddin et al., 2014), GC-MS (Huang et al., 2015), and ultra-HPLC (UHPLC) coupled MS/MS spectrometry (Floková et al., 2014), have been previously reported for SA detection. Capillary electrophoresis (CE) is an alternative to HPLC-based methods by virtue of the high separation efficiency and low solvent consumption advantages. Lin's group designed an iron oxide-based solid phase extraction system coupled to CE-UV for analyzing SA (Chang et al., 2017). This method shows excellent sensitivity for SA detection. Furthermore, there was an electrochemical sensor developed for determination of SA recently, in order to improve the sensitivity and accuracy, the composite of multi-wall carbon nanotubes (MWNT) and carbon black (CB) was fabricated, and ferrocene (Fc) was exploited as the reference molecule to offer a built-in correction (Hu et al., 2020). In addition, a work from Wang and co-workers reported that a nanosensor had been prepared based on the structure-switching strategy, and the SA aptamer showed a good affinity to SA (Chen et al., 2019).

In comparison with other methods, the colorimetric method has garnered extensive attention due to its superior responsiveness, portability, and practicality with no need for expensive and cumbersome instruments (Yan et al., 2020). Additionally, the detection result is easy to read out with visual inspection (Hao et al., 2013; Fan et al., 2017). In some research, a colorimetric assay has been developed for the detection of SA using TiO_2_ NPs. This nanomaterial shows good selectivity for SA, however, the sensitivity of detection is limited (Tseng et al., 2014). In another report, Yang's group developed two rhodamine-based fluorescent probes that could realize bioimaging of SA (Wang et al., 2019a). This colorimetric sensor not only possesses good selectivity and sensitivity toward SA but detection is also observable with the naked-eye. At the same time, it has some shortcomings. The method is time-consuming, involves fussy operation steps, and it is difficult to fabricate probes using it. Previous studies have made it clear that nanozymes can be used in the field of colorimetric sensing owing to the advantages of simple preparation, low cost, and high catalytic activity. In particular, based on the improvement of enzyme catalytic activity, many studies have been carried out to achieve the purpose of colorimetric detection (Zhang et al., 2018; Li et al., 2019). Besides, most studies have focused on the design of nanozymes with different activities for colorimetric sensing of biothiols (Xiong et al., 2015; Song et al., 2020), dopamine (Wang et al., 2019b), glutathione reductase (Zhang et al., 2019), and acid phosphatase (Lin et al., 2020). These sensors generally achieve the goal of detection by using reduced substances to interfere with the oxidation of chromogenic substrates or to reduce oxTMB. However, these methods are usually affected by substances with strong reducibility, and thus the samples generally need to be pretreated. In contrast, few reports have been devoted to designing a scheme for specifically inhibiting the activity of nanozymes to ensure the selectivity of colorimetric sensing.

**Figure 1:**
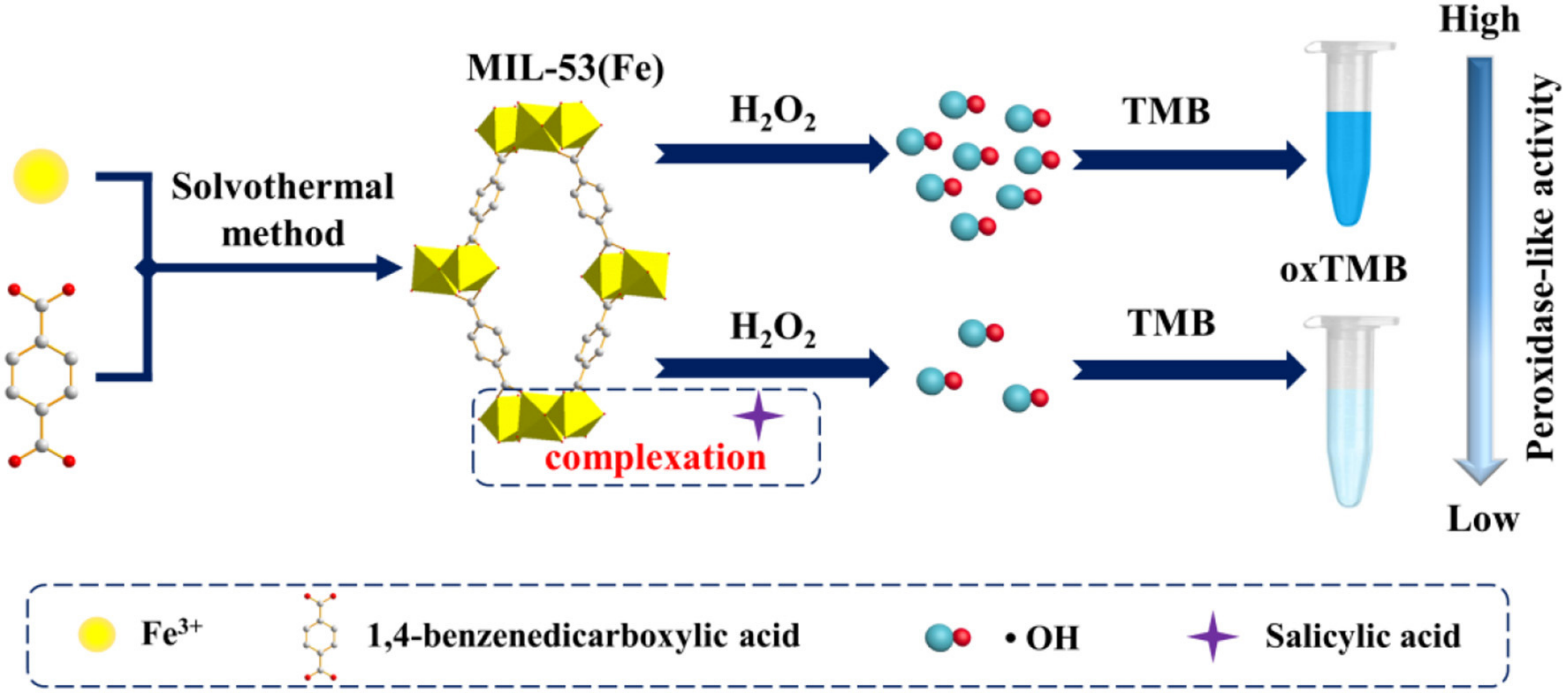
Schematic illustration of MIL-53(Fe) synthesis and colorimetric detection of SA based on the MIL-53(Fe) nanozyme.

Thus, herein, inspired by the specific complexation reaction between SA and Fe^3+^, and considering that no efforts have been made toward colorimetric detection of SA in aspirin by utilizing a metal-organic framework (MOF) nanozyme. we select MIL-53(Fe) as the nanozyme serving to achieve colorimetric detection of SA (Figure 1). As a result, the as-prepared MIL-53(Fe) shows high peroxidase-like activity in catalyzing 3,3,5,5-tetramethylbenzidine (TMB) oxidation and thus makes it a blue color in the presence of H_2_O_2_. Interestingly, because of the complexation reaction of SA and Fe^3+^ in the center of MIL-53(Fe), the catalytic activity of MIL-53(Fe) is inhibited significantly with the addition of SA. Based on this, a simple colorimetric sensor was successfully developed to detect SA. The proposed system furnishes a cost-effective, highly selective, and sensitive strategy for the colorimetric detection of SA and broadens the application potential of MOF nanozymes in the field of pharmaceutical analysis.

## Experimental

### Materials

Acetic acid (HAc), H_2_O_2_ (30 wt%), *N,N'*-dimethylformamide (DMF), sodium acetate (NaAc), salicylic acid (SA), and 1,4-benzenedicarboxylic acid (H_2_BDC) were acquired from Beijing HWRK Chem Co., LTD. (China). Iron chloride hexahydrate (FeCl_3_·6H_2_O) and TMB were bought from Shanghai Macklin Biochemical Co., Ltd. (China). All the chemicals mentioned above were analytical grade and used as received. Aspirin was purchased from Shanghai Titan Scientific Co., Ltd. Ultra-pure water came from the Millipore purification system (18.2 MΩ).

### Apparatus

The powder X-ray diffraction (XRD) pattern of MIL-53(Fe) was recorded with a D/max 2,550 VB/PC diffractometer (Rigaku, Japan) using Cu Kα radiation (λ = 0.15418 nm). Scanning electron microscopy (SEM) and transmission electron microscopy (TEM) pictures were taken using Quanta 200 FEG (Netherlands) and Tecnai G2 20 (PE, USA), respectively. A Fourier transform infrared (FTIR) spectra was obtained from spectrum-2000 (USA). Thermogravimetric analysis (TGA) was carried out by the LABSYS evo TG-DSC/DTA instrument (Setaram Instrumentation, France). The UV-Vis absorption spectrum was recorded on the Cary 60 spectrophotometer (Agilent, USA). And HPLC was performed on the liquid chromatography instrument (Shimadzu, Japan) with the LC-20AT pump and the SPD-20A UV-vis detector.

### Preparation of MIL-53(Fe)

MIL-53(Fe) was synthesized with a solvothermal method referring to the literature with a minor modification (Millange et al., 2010). Typically, 1.0812 g FeCl_3_·6H_2_O was dissolved in 20 mL of DMF solution before adding 0.6646 g of H_2_BDC. After 10 min of intense stirring, the solution was transferred to a 50 mL stainless-steel autoclave and then heated at 150°C for 48 h. After natural cooling, the yellow precipitate was collected by centrifugation and washed with distilled water and ethanol. Finally, the yellow precipitate was dried in vacuum at 60°C overnight.

### Detection of SA

To obtain a standard curve, SA detection was conducted as follows: 70 μL 1 mg/mL MIL-53(Fe) and 25 μL aliquots of SA solution of different concentrations were added to 875 μL 0.1 M pH 3.5 acetate buffers. After incubating for 4 min, 30 μL 4 mM TMB and 25 μL 0.1 M H_2_O_2_ were added to the above solution, and the solution was shaken and incubated at 37°C for 8 min. Finally, a Cary 60 spectrophotometer was used for analyzing the mixed solution.

### Analysis of SA in Aspirin Sample

A standard addition method was applied for the determination of SA in aspirin. The sample was prepared as follows: 100 mg of aspirin powder was dissolved in 1% glacial acetic acid methanol solution (100 mL) and then filtered through a 0.45 μm filter paper. Next, the filtrate (100 μL) was placed in 0.1 M pH 3.5 acetate buffers. Subsequently, 70 μL 1 mg/mL of MIL-53(Fe) and different concentrations of SA were added. After incubating for 4 min, 30 μL 4 mM TMB and 25 μL 0.1 M H_2_O_2_ were added. At last, the mixture was incubated at 37°C for 8 min, the resulting solution was analyzed by the proposed colorimetric method, and the corresponding recoveries were obtained.

## Results and Discussion

### Characterization of MIL-53(Fe)

**Figure 2:**
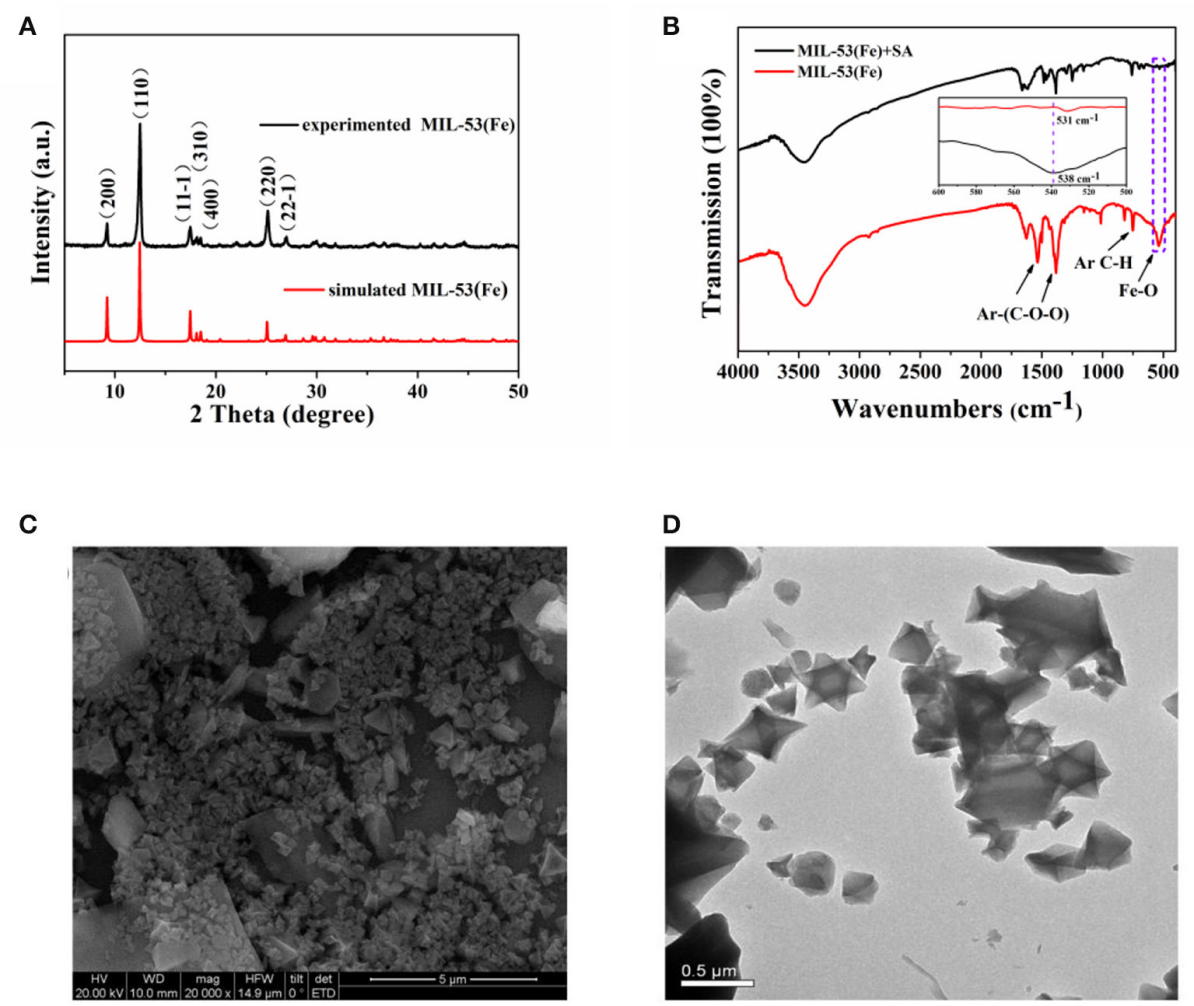
**(A)** PXRD patterns of the as-prepared MIL-53(Fe) (black curve) and the simulated MIL-53(Fe) (red curve), **(B)** FTIR spectra of MIL-53(Fe) and MIL-53(Fe)+SA, **(C,D)** depicting the SEM and TEM images of MIL-53(Fe), respectively.

The structure of MIL-53(Fe) was characterized first by XRD. As Figure 2A shows, particles of MIL-53(Fe) are highly crystalline, and the obvious sharp diffraction peaks correspond to the previously reported results as well as the simulated results (Lin et al., 2018). In addition, FTIR spectra was utilized for identifying the characteristic functional groups. As can be seen from Figure 2B, the spectrum clearly exhibits the typical asymmetrical and symmetrical vibration bands of carboxyl groups on the ligand, which indicates the existence of a dicarboxylate linker in MIL-53(Fe). Meanwhile, the C-H bending vibration of the benzene ring is observed and the stretching vibration of the Fe-O bond well-explains the formation of the metal-oxo cluster between Fe(III) and the carboxylic group of the organic linker. However, in the presence of SA, the absorption peak of the Fe-O bond shifted from 538 to 531 cm^−1^, this result indicates that SA reacts with MIL-53(Fe) to form an iron salicylate complex. Furthermore, the morphology of MIL-53(Fe) was characterized by SEM and TEM. As shown by the images, the particles of MIL-53(Fe) have a very regular octahedron crystal structure (Figures 2C,D). Afterwards, the mass loss was explored via thermogravimetric analysis (TGA), it was ascertained that the first mass loss (~39.27%) was due to the dehydration of MIL-53(Fe), and the subsequent mass loss (~56.56%) corresponded to the collapse of MIL-53(Fe) into Fe_2_O_3_. The result from Figure S1 is well-within the expected range according to the previous report (Millange et al., 2010). It could be concluded that MIL-53(Fe) was synthesized successfully with overall consideration in the characterization results above.

### Detection Principle

In recent years, MIL-53(Fe) has been demonstrated exhibiting intrinsic peroxidase-mimicking catalytic activity and has been used for the colorimetric detection of ascorbic acid (AA) (Ai et al., 2013). In the presence of H_2_O_2_, the chromogenic substrates are oxidized with the catalytic effect of the MIL-53(Fe) nanozyme. As a result, the color of the solution is changed. In this work, a lighter color of oxTMB is created due to the inhibitory effect of SA on MIL-53(Fe) activity. Aiming at this phenomenon, a facile colorimetric platform was established to monitor SA (Figure 1). During the experiment, MIL-53(Fe) and SA were incubated in an acetate buffer for 4 min, then TMB and H_2_O_2_ were added and the mixture was incubated at 37°C for 8 min. In the absence of SA, MIL-53(Fe) maintains its catalytic activity and a deep blue color for oxTMB emerges in the MIL-53(Fe)/TMB/H_2_O_2_ system. On the contrary, SA has strong complexation ability with Fe^3+^ in the center of MIL-53(Fe). After the addition of SA, the peroxidase-like activity of MIL-53(Fe) is significantly inhibited due to the reaction of SA and Fe^3+^ (Figure 1). Consequently, the catalytic oxidation of TMB begins to decelerate, enabling the blue color solution to lighten. The color change is easy to identify by the naked eye. The catalytic activity of MIL-53(Fe) and the absorption intensity of oxTMB at 652 nm are dependent on the SA concentration. Thus, the MIL-53(Fe)/TMB/H_2_O_2_ system is capable for the colorimetric detection of SA.

**Figure 3:**
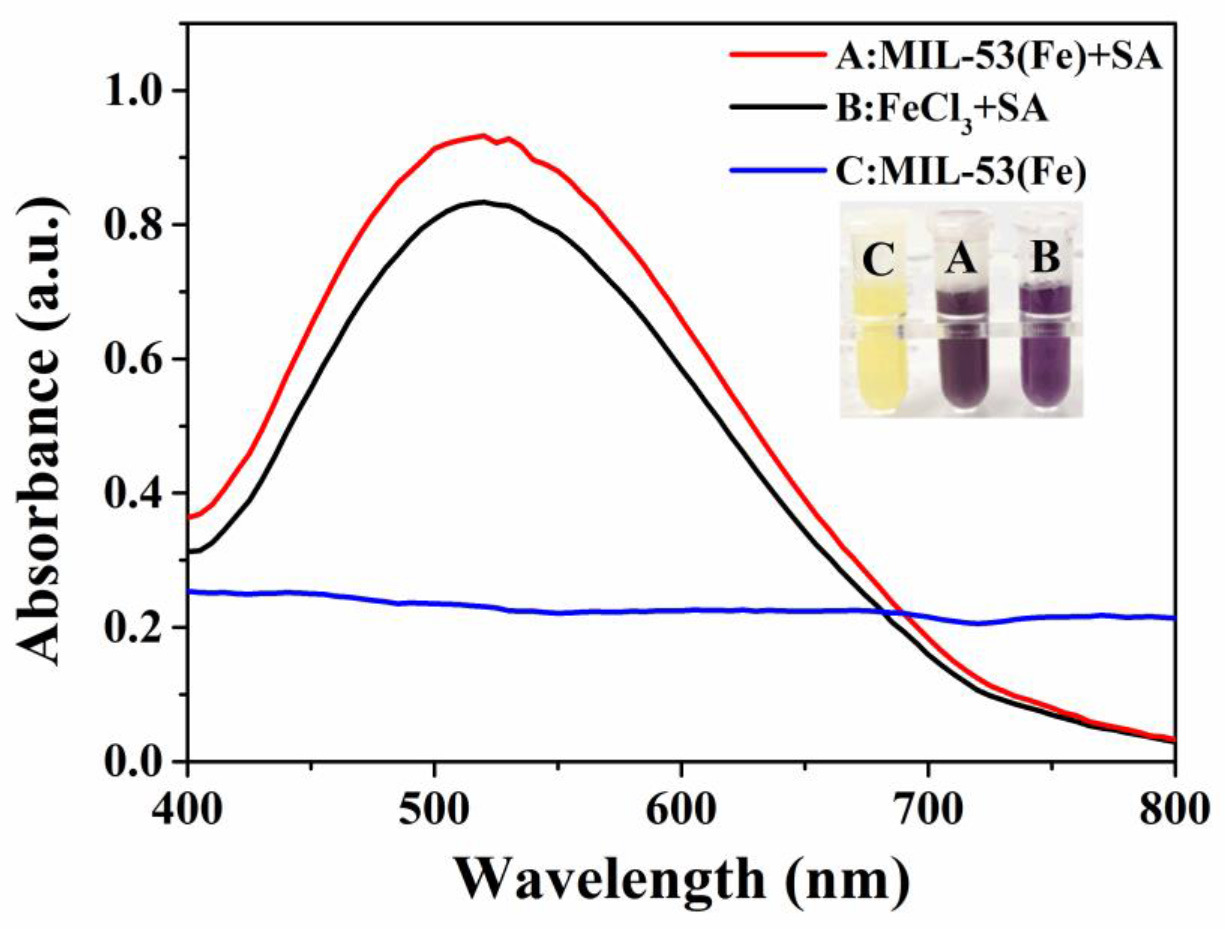
The absorption peaks of SA-Fe(III) complex characterized by UV-Vis spectra: **(A)** MIL-53(Fe)+SA, **(B)** FeCl_3_+SA, **(C)** MIL-53(Fe). Inset: the corresponding photograph of different systems.

To illuminate the mechanism of inhibitory effects of SA on the MIL-53(Fe) activity, the purple complex of SA-Fe(III) was characterized by UV-Vis absorbance spectroscopy. As revealed by Figure 3, a conspicuous absorption peak at ~520 nm appears when FeCl_3_ is used as the standard sample to incubate with SA (Chang et al., 2017; Bodek et al., 2020), and the color of the solution changes to purple. Likewise, the same absorption peak at 520 nm occurs when MIL-53(Fe) and SA are incubated together. The solution of MIL-53(Fe) is yellow and has turned a dark purple color with the addition of SA. Obviously, no peak appears at 520 nm when there is only MIL-53(Fe) in the solution. What this suggests is that Fe(III) in the center of MIL-53(Fe) forms a purple complex with SA and results in the inhibited activity of MIL-53(Fe).

To further demonstrate the inhibitory effect of SA on the peroxidase-like activity of MIL-53(Fe), the production of hydroxyl radical (·OH) within the reaction was investigated. Electron spin resonance (ESR) spectroscopy was carried out with 5,5-dimethylpyrroline N-oxide (DMPO) as the ·OH trapping agent (Zhao et al., 2019). As depicted in Figure S2, it shows typical ·OH signals in the MIL-53(Fe)+H_2_O_2_ system. However, the signal intensity is obviously decreased with the addition of SA. To be specific, as Fe^3+^ ions in the center of MIL-53(Fe) are Fenton-like reagents, they can catalyze the decomposition of H_2_O_2_ into ·OH via a Fenton-type reaction (Yao et al., 2018). In the meantime, the redox cycle of Fe^3+^/Fe^2+^ is decelerated due to the complexation of SA to Fe^3+^. Consequently, the production of hydroxyl radical (·OH) in the reaction is decreased. This also proves that the catalytic activity of MIL-53(Fe) is inhibited by SA.

### Feasibility of SA Detection by MIL-53(Fe) Nanozyme

**Figure 4:**
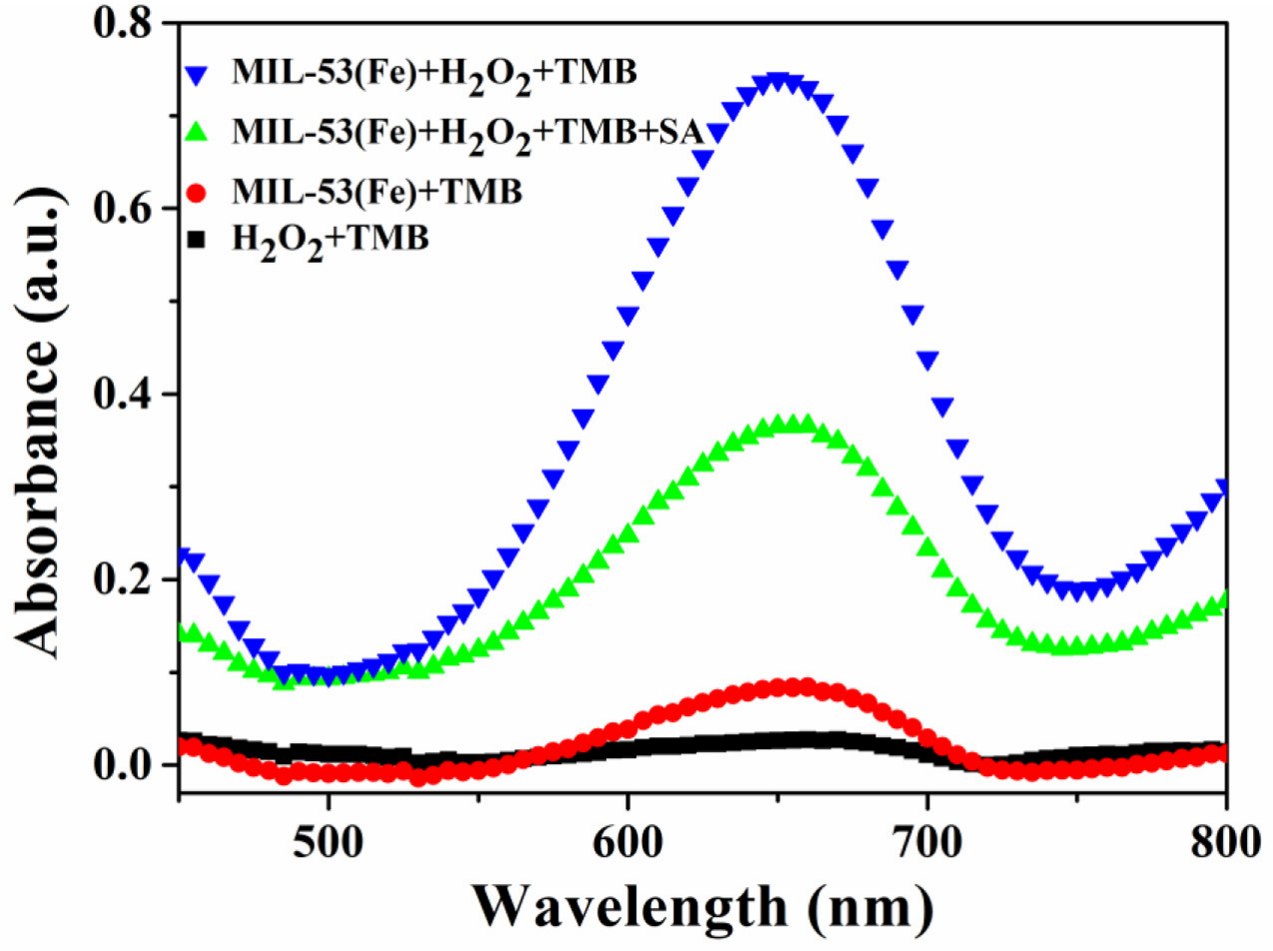
UV-Vis absorbance spectra on the feasibility analysis for the detection of SA based on the MIL-53(Fe) nanozyme.

A feasibility test for SA detection was performed and the result is displayed in Figure 4. Obviously, the highest absorption peak occurs when MIL-53(Fe), H_2_O_2_, and TMB are mixed and incubated together, this is ascribed to the potential peroxidase-like activity of the as-prepared MIL-53(Fe). Additionally, no obvious absorption peak can be observed during either of the two groups (the group of H_2_O_2_+TMB or MIL-53(Fe)+TMB). What is interesting, however, is that while SA is added to the MIL-53(Fe)/TMB/H_2_O_2_ system, the absorption peak intensity is decreased significantly. As we know, SA has good strong complexation ability to Fe^3+^ ions, thereby forming iron salicylate complexes. That might be the reason why SA has an effective inhibitory effect on the catalytic activity of MIL-53(Fe). In brief, the proposed colorimetric method is built on the inhibitory effect of SA on the inherent activity of the MIL-53(Fe) nanozyme.

### Optimization of Experimental Conditions

**Figure 5:**
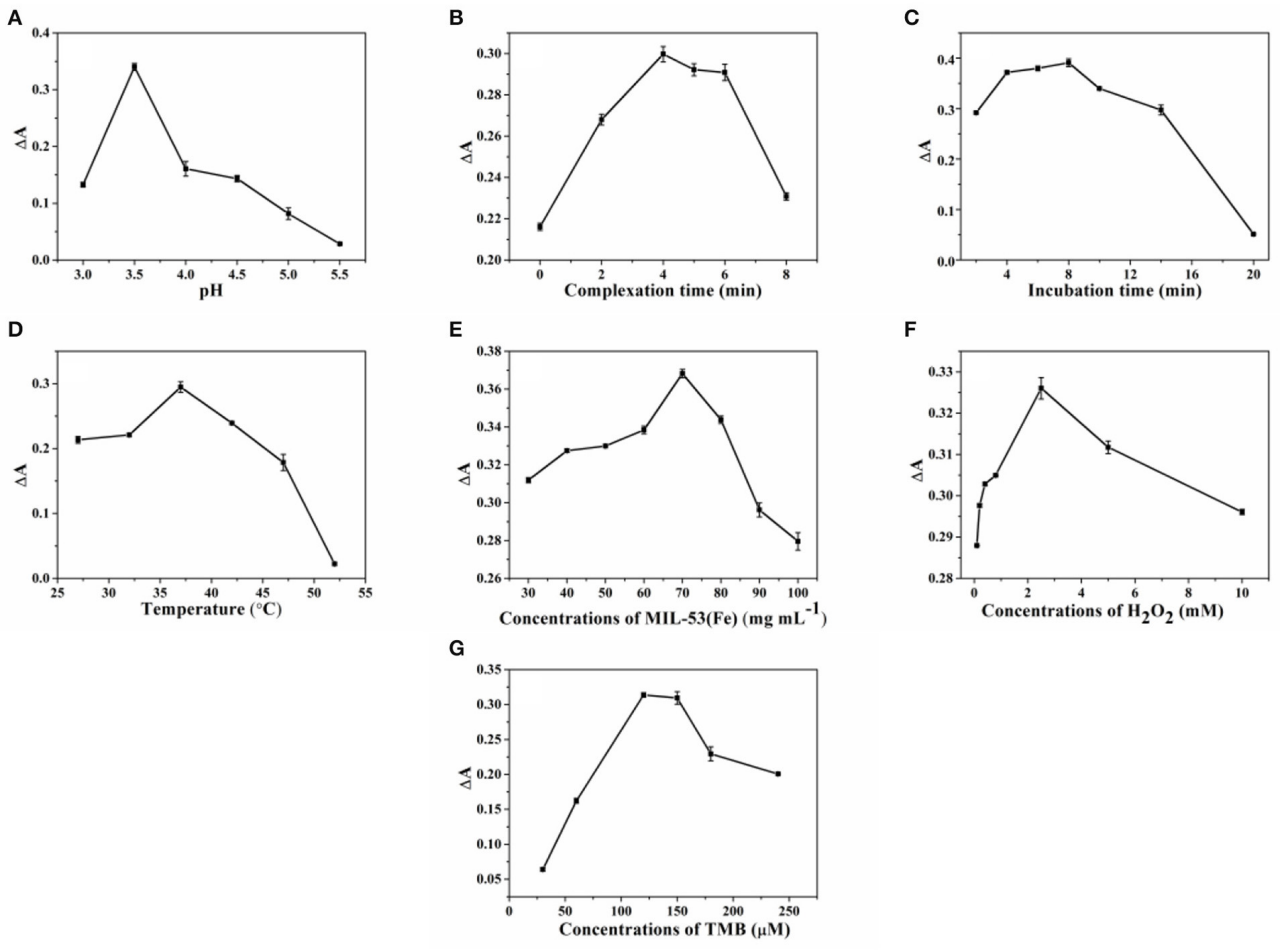
The optimization of **(A)** pH, **(B)** complexation time, **(C)** incubation time, **(D)** temperature, and concentrations of **(E)** MIL-53(Fe), **(F)** H_2_O_2_, and **(G)** TMB for SA sensing. Error bars represent the standard deviation of the three trials.

In realizing the best performance in SA detection, the influence of the experimental conditions, such as pH, complexation time, incubation time, temperature, and concentrations of MIL-53(Fe), H_2_O_2_, and TMB, were investigated. Since the activity of MIL-53(Fe) and the reaction of MIL-53(Fe) with SA are closely related to the pH value, therefore the effects of different pH values (3.0, 3.5, 4.0, 4.5, 5.0, and 5.5) were first studied. ΔA (A-A_0_, A, and A_0_ represent the absorbance of the system without or with SA, respectively), is used as the index to evaluate the best conditions for the detection of SA. As can be seen from Figure 5A, it is clear that the value of ΔA reaches a peak at pH 3.5. This can be explained by the fact that the activity of MIL-53(Fe) and the complexation ability of MIL-53(Fe) with SA is higher at pH 3.5. Consequently, 3.5 was chosen as the optimal pH value. Figures 5B,C clearly show that along with the increase of reaction time, the absorbance intensity (ΔA) starts to decrease after 4 and 8 min, indicating that 4 min was the most satisfactory time for MIL-53(Fe) to bind with SA and 8 min was the most suitable incubation time for the experiment. Moreover, the optimal temperature from 27 to 52°C was further discussed. As seen from Figure 5D, the value of ΔA declined dramatically as the temperature reached 37°C, hence, 37°C was considered as the optimal temperature for the reaction. Furthermore, the concentration of MIL-53(Fe) plays a key role in its catalytic activity. Figure 5E exhibits that with an increasing MIL-53(Fe) concentration, the ΔA value increased gradually until 70 μg L^−1^, showing that the concentrations of 70 μg L^−1^ of MIL-53(Fe) is sufficient for detecting SA. Additionally, Figures 5F,G depicted that TMB and H_2_O_2_ concentrations have an effect on the absorbance intensity. The value of ΔA reached the top as the concentrations of TMB and H_2_O_2_ increased to 120 μM and 2.5 mM, respectively. Overall, after optimization, 3.5, 4 min, 8 min, 37°C, 70 μg L^−1^, 120 μM, and 2.5 mM are shown to be the optimal pH value, complexation time, incubation time, temperature, and concentrations of MIL-53(Fe), H_2_O_2_, and TMB.

### Colorimetric Detection of SA

**Figure 6:**
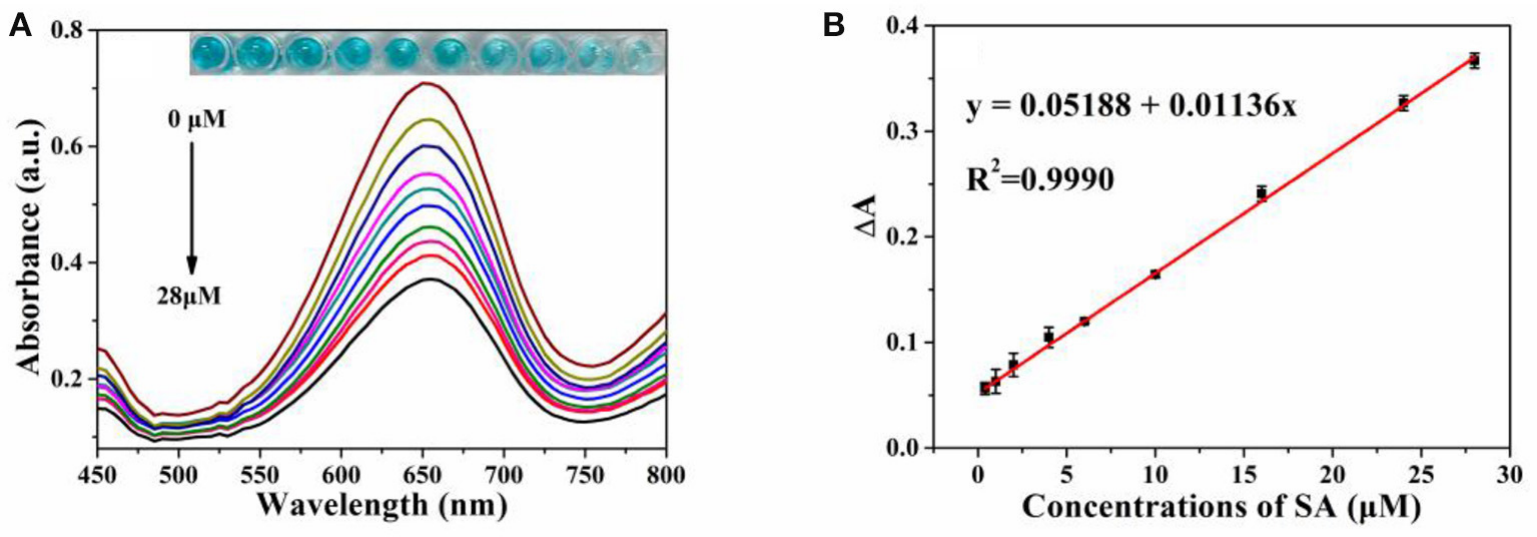
**(A)** The vis absorption spectra of MIL-53(Fe)-TMB-H_2_O_2_ upon concentrations of SA (from 0 to 28 μmol L^−1^). Inset: the corresponding photograph of the colored products; **(B)** The linear relationship between absorbance intensity (ΔA) and SA level. Error bars represent the standard deviation of three trials.

Since the addition of SA can significantly inhibit the peroxidase-like activity of MIL-53(Fe), a colorimetric assay for the determination of SA was developed. Under the optimal experimental conditions, different concentrations of the SA solution were added to the MIL-53(Fe)/TMB/H_2_O_2_ system. Meanwhile, the mixed solution was detected and the obtained absorption spectrum was analyzed. Eventually, the limit of detection (LOD) was calculated by using the signal-to-noise ratio (S/N = 3) (He et al., 2018). As revealed by Figure 6A, absorbance intensities at 652 nm decreased gradually as the concentrations of SA increased from 0 to 28 μmol L^−1^, and the color change of oxTMB is visible to the naked eye. In the meantime, as depicted in Figure 6B, there was an excellent linear relationship between absorbance intensity (ΔA) and SA concentrations in the range of 0.4–28 μmol L^−1^ (*R*^2^ = 0.9990), and the LOD was calculated to be 0.26 μmol L^−1^. The Chinese Pharmacopeia (edition 2015), set a standard that the maximum limit of SA in aspirin is 0.3% (2.16 μmol L^−1^). Hence, the value of LOD (0.26 μmol L^−1^) was lower than the safety limits. Furthermore, the LOD value and linear ranges of our method are compared with those of other methods. As shown in Table S1, the sensitivity of our measurement was superior to most previously reported values, indicating that the established method was appropriate for SA detection.

### Selectivity of the Colorimetric Biosensor

**Figure 7:**
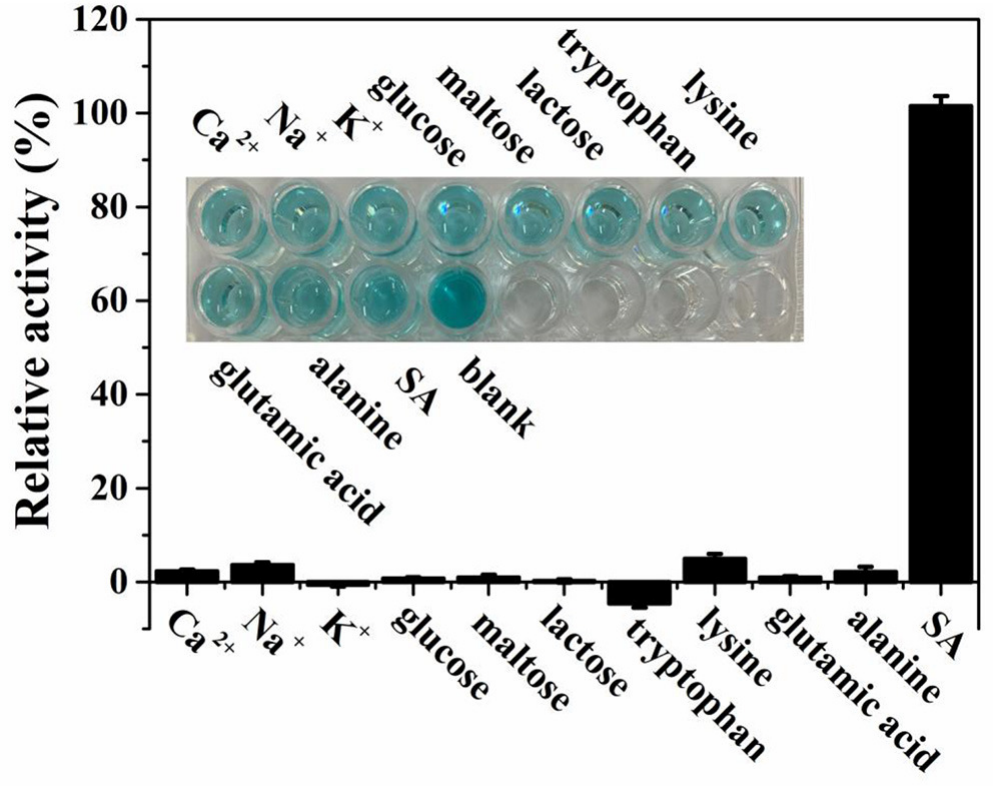
Selectivity for SA detection with existence of several possible coexisting substances based on theMIL-53(Fe)-TMB-H_2_O_2_ system. The inset shows the corresponding photograph of the interference experiment.

In order to study the selectivity of our assay toward SA detection, possible coexisting substances including some metal ions, amino acids, and carbohydrates were used for investigating under the optimal experimental conditions. As demonstrated in Figure 7, obviously, only the species SA can induce a significant absorption intensity when the concentrations of SA and possible coexisting substances are both 28 μmol L^−1^. Additionally, as illustrated in the inset of Figure 7, there was no interference with the color change in the presence of other coexisting substances. All the results above show that the biosensor designed for colorimetric detection of SA was highly selective.

### Detection of SA in Aspirin Samples

To confirm the reliability of the developed platform for colorimetric detection of SA, the original amount of SA in aspirin was first measured by using the HPLC method (Figure S3), and the detection results were compared with those obtained by colorimetry. As displayed in Table 1, the detected SA concentrations by the proposed strategy have shown satisfactory agreement in comparison with the measured data from HPLC, the relative error was within 5%. Meanwhile, to further investigate the precision and practicability of our assay, a spike recovery test was carried out. As demonstrated by Table S2, it shows that a meaningful recovery of 91.0–101.4% was obtained and the value of RSD ranged from 2.0 to 4.2%. In a word, the results proved the potential of this proposed method in practical applications.

**Table 1:** The recovery test of our assay for detecting SA in Pharmaceutical samples.

**Sample**	**Original amount (μM)**	**Spiked (μM)**	**Found (μM)**	**Recovery (%)**	**RSD (%, *n* = 3)**
Sample 1	1.55	12	10.92	91.0	4.2
		20	18.61	93.0	3.8
Sample 2	2.37	10	9.97	99.7	3.0
		14	13.7	98.0	4.0
Sample 3	3.83	8	8.11	101.4	2.0
		16	15.39	96.2	3.5

## Conclusions

In this study, we confirmed that the obtained MIL-53(Fe) possessed inherent peroxidase-mimicking activity, as they were equipped to catalyze the oxidation of chromogenic TMB in the presence of H_2_O_2_. Meanwhile, based on the strong complexing action between the Fe^3+^ in the center of MIL-53(Fe) and SA, the activity of MIL-53(Fe) can be effectively inhibited with the addition of SA, causing fewer hydroxyl radicals to be produced in the system to decelerate the oxidation of TMB. The experimental results clearly displayed that a lighter color for the oxTMB can be observed with naked eye. By combining the peroxidase-mimicking properties of MIL-53(Fe) and the inhibitory effect of SA on its activity, a colorimetric sensing platform for the detection of SA was established. The developed colorimetric method not only has excellent accuracy in detecting SA in aspirin compared with HPLC but also shows high selectivity and sensitivity to SA. In summary, the proposed method can be well-utilized for detecting SA in aspirin. Furthermore, the present work displays the great potential of using MOFs nanozymes for pharmaceutical analysis.

## Data Availability Statement

The raw data supporting the conclusions of this article will be made available by the authors, without undue reservation.

## Author Contributions

LL: conceptualization, methodology, data curation, investigation, and writing original draft. YH: conceptualization, methodology, data curation, and investigation. WL: data curation and investigation. WZ: investigation. FY: visualization, resources, funding acquisition, supervision, project administration, writing—review, and editing. SZ: funding acquisition and project administration. All authors contributed to the article and approved the submitted version.

## Conflict of Interest

The authors declare that the research was conducted in the absence of any commercial or financial relationships that could be construed as a potential conflict of interest.
