# Supplementary material for: Colorimetric Detection of Salicylic Acid in Aspirin Using MIL-53(Fe) Nanozyme
Source: Front Chem. 2020 Sep 18;8:671. doi: 10.3389/fchem.2020.00671 (PMC7530239; doi:10.3389/fchem.2020.00671)
Supplement: Supplementary file 1 [file Data_Sheet_1.docx]

**Figure and Table captions**

**Fig. S1** TGA curve of MIL-53(Fe).

**Fig. S2** ESR spectrum of different groups treated with DMPO.

**Fig. S3** The standard curve of SA concentrations and peak area.

**Table S1** Comparison of analysis parameters about different methods for SA detection.

**Table S2.** Recovery Test of SA in Pharmaceutical samples.

**Fig. S1**





**Fig. S2**





**Fig. S3**





**Table S1.** Comparison of analysis parameters about different methods for SA detection

| **Method** | **Materials** | **Linear**  **Range (μM)** | **LOD**  **(μM)** | **Detection**  **conditions** | **Application** | **References** |
| --- | --- | --- | --- | --- | --- | --- |
| Chromatography | / | 0.72-28.99 | 0.22 | EtOH/H_2_O (3/1, v/v) and NaOH | Plant leaves | [S1] |
| Fluorescent | Curcumin-Cu^2+^ | 10-150 | 3 | Neutral or weakly alkaline | In vitro  and cells | [S2] |
| Electrochemical | Screen printed electrodes | 16-300 | 5.6 | pH 7, 22℃±2℃ | Human urine | [S3] |
| Electrochemical | Modified CB-MWNT-nafion/Fc/CBMWNT/GC electrode | 25-1000 | 3.3 | pH 7.4 | Soybean seedlings | [S4] |
| Colorimetric | TiO_2_ NPs | 20-1000 | 15.4 | pH 5.5, 25℃ | Tobacco leaves | [S5] |
| Colorimetric | MIL-53(Fe) | 0.4-28 | 0.26 | pH 3.5, 37℃ | Aspirin | This work |

**Table S2.** Recovery test of SA in pharmaceutical samples

| **Sample** | **HPLC**  **(μM)** | **This method**  **(μM)** | **Relative**  **error (%)** | **RSD**  **(%, n=3)** |
| --- | --- | --- | --- | --- |
| **Sample 1** | 1.60 | 1.55 | 3.4 | 1.3 |
| **Sample 2** | 2.26 | 2.37 | 4.8 | 2.5 |
| **Sample 3** | 3.69 | 3.83 | 3.8 | 1.7 |

[S1] T. L. Marques, L. M. B. Moraes, F. R. P. Rocha, Systematic evaluation of sample preparation for fractionation of phytohormone salicylic acid in fresh leaves, *Talanta*, 2020, 208, 120352.

[S2] C. Chen, L. L. Yang, A. L. Tang, P. Y. Wang, R. Dong, Z. B. Wu, Z. Li, S. Yang, Curcumin-Cu (II) ensemble-based fluorescence “turn-On” mode sensing the plant defensive hormone salicylic acid in situ and in vivo, *J. Agric. Food Chem.*, 2020, 68, 4844-4850.

[S3] S. Rawlinson, A. McLister, P. Kanyong, J. Davis, Rapid determination of salicylic acid at screen printed electrodes, *Microchem. J.*, 2018, 137, 71-77.

[S4] Y. Hu, X. D. Wang, C. Wang, P. C. Hou, H. T. Dong, B Luo, A. X. Li, A multifunctional ratiometric electrochemical sensor for combined determination of indole-3-acetic acid and salicylic acid, *RSC. Adv.*, 2020, 10, 3115-3121.

[S5] P. J. Tseng, C. Y. Wang, T. Y. Huang, Y. Y. Chuang, S. F. Fu, Y. W. Lin, A facile colorimetric assay for determination of salicylic acid in tobacco leaves using titanium dioxide nanoparticles, *Anal. Methods.*, 2014, 6, 1759-1765.
